# Diagnostic Discrimination of BOKE STARS, a Bimodal Continuous Performance Test, for Attention-Deficit/Hyperactivity Disorder Assessment in Chinese Children: Single-Center Case-Control Study

**DOI:** 10.2196/82164

**Published:** 2026-07-21

**Authors:** Yangyang Xu, Xiaoqian Li, Meiling Cao, Jing Li, Shuneng Gu, Lin Lv, Heying Zhang, Jianjuan Zhao, Bin Li, Jian Li, Feng Li

**Affiliations:** 1 Department of Ophthalmology, Shanghai Jiao Tong University, School of Medicine, Xinhua Hospital, 1665 Kongjiang Road, Shanghai, Yangpu District, 200092, China, +86-21-25078505; 2 Department of Developmental and Behavioral Pediatrics, Shanghai Jiao Tong University, School of Medicine, Shanghai Children’s Hospital, Shanghai, China; 3 BOKE Digital Health Research Institute, BOKE Medical Technology (Shanghai) Co., Ltd., BOKE Technology Group, Shanghai, China; 4 Department of Developmental Behavioral Pediatric and Children Healthcare, Shanghai Jiao Tong University, School of Medicine, Xinhua Hospital, 1665 Kongjiang Rd, Shanghai, 200092, China, 86 13512111965

**Keywords:** attention-deficit/hyperactivity disorder, ADHD, continuous performance test, diagnostic accuracy, assessment, China

## Abstract

**Background:**

Attention-deficit/hyperactivity disorder (ADHD) affects 6.3% of Chinese children, but only 10% are diagnosed, as diagnosis is hindered by low awareness and lack of culturally adapted, objective tools. Existing continuous performance tests are unimodal and lack validity.

**Objective:**

This study aimed to conduct an initial evaluation of the diagnostic discrimination of BOKE STARS (Sustained Task and Attention Response Screening), a culturally adapted bimodal continuous performance test, for distinguishing children with ADHD from typically developing children in a Chinese clinical setting.

**Methods:**

In this prospective, single-center diagnostic accuracy study with a case-control design, 100 children aged 6 to 12 years (n=50 with ADHD and n=50 controls) were recruited at Xinhua Hospital between January and May 2024. Parents completed the Swanson, Nolan, and Pelham Rating Scale–fourth version (SNAP-IV), and children completed the BOKE STARS assessment on a tablet device under standardized conditions. Group comparisons were conducted using independent-sample 2-tailed *t* tests or Mann-Whitney *U* tests, as appropriate. Receiver operating characteristic (ROC) curve analysis was performed in the full case-control sample to assess diagnostic discrimination. Sensitivity and specificity were reported descriptively across ROC-derived cutoffs; these cutoffs were not interpreted as clinically validated diagnostic thresholds. Secondary exploratory analyses included comparisons among ADHD subtypes and correlations between BOKE STARS indices and parent-reported SNAP-IV symptom severity scores.

**Results:**

Compared with controls, children with ADHD performed significantly worse on all major BOKE STARS indices, including errors of omission, errors of commission, reaction time, reaction time variability (RTV), discrimination prime, and total score. In the full case-control sample, the total score showed the strongest diagnostic discrimination (area under the ROC curve [AUC] 0.962, 95% CI 0.931-0.992), followed by RTV (AUC 0.919, 95% CI 0.867-0.971), errors of omission (AUC 0.884, 95% CI 0.818-0.950), and discrimination prime (AUC 0.819, 95% CI 0.737-0.900). Errors of commission (AUC 0.689, 95% CI 0.585-0.792) and reaction time (AUC 0.634, 95% CI 0.524-0.743) showed comparatively weaker discrimination. No significant differences were observed among ADHD subtypes. Several BOKE STARS indices were modestly correlated with SNAP-IV inattention and hyperactivity or impulsivity scores.

**Conclusions:**

BOKE STARS showed promising preliminary diagnostic discrimination for identifying Chinese children with ADHD in this case-control sample, with the total score and RTV showing the strongest discriminatory performance. However, because the case-control design artificially fixed the ratio of ADHD cases to controls, diagnostic performance estimates and exploratory cutoffs should be interpreted cautiously and should not be considered representative of real-world clinical diagnostic performance. BOKE STARS may serve as an adjunctive assessment tool to complement clinical interviews and caregiver-reported rating scales, but further external validation in larger and clinically heterogeneous populations is required before broader clinical implementation.

## Introduction

Attention-deficit/hyperactivity disorder (ADHD) is a common neurodevelopmental disorder of childhood that often persists into adolescence and adulthood and is associated with a substantial functional burden across academic, social, and family domains [1,2]. In China, the pooled prevalence of ADHD in children and adolescents has been estimated at 6.26%, indicating a large affected population and a substantial public health need for timely identification and intervention [3].

Current ADHD diagnosis still relies primarily on clinical interviews, behavioral history, and standardized rating scales. Although these approaches remain central to clinical decision-making, they are inevitably influenced by informant perspectives, clinical experience, and the availability of specialist resources [4]. As a result, there is sustained interest in performance-based tools that can provide more objective behavioral information to support the clinical assessment process.

Continuous performance tests (CPTs) are among the most widely used computerized paradigms for assessing sustained attention, response inhibition, and reaction time stability. Commonly used systems such as the Conners CPT and the Test of Variables of Attention have been studied extensively in ADHD and typically quantify omission errors, commission errors, reaction time, and reaction time variability (RTV) [5-7]. However, recent evidence suggests that the clinical utility of CPTs, when used in isolation, remains limited and that they should not be interpreted as standalone diagnostic tools [7-11].

In Chinese clinical settings, the application of traditional CPT tools may also be constrained by language, cultural adaptation, and practical implementation issues. At the same time, most commonly used CPTs rely on unimodal visual or auditory stimuli, whereas emerging evidence suggests that bimodal paradigms may improve signal detection and discriminatory performance in ADHD-related attention testing [7,12-14]. These considerations support the development of locally adapted CPT tools that are more suitable for Chinese children and more feasible for real-world clinical use. The Swanson, Nolan, and Pelham Rating Scale–fourth version (SNAP-IV) is a widely used caregiver-reported rating scale for assessing ADHD-related inattentive and hyperactive or impulsive symptoms, as well as oppositional defiant behaviors. The Chinese parent version of the SNAP-IV has demonstrated acceptable psychometric properties and has been used in Chinese clinical and research settings [15].

BOKE STARS (Sustained Task and Attention Response Screening) is a tablet-based bimodal audiovisual CPT developed for Chinese pediatric users. It uses synchronized visual and auditory stimuli and generates standardized performance indices, including omission errors, commission errors, reaction time, RTV, discrimination prime, and total score. In this study, we conducted an initial evaluation of the diagnostic discrimination of BOKE STARS in distinguishing children with ADHD from typically developing (TD) controls in a Chinese clinical setting. Secondary exploratory aims were to compare BOKE STARS indices across ADHD subtypes and to examine correlations between BOKE STARS indices and parent-reported SNAP-IV symptom severity scores.

## Methods

### Study Design

This study was designed as a prospective, single-center diagnostic accuracy study with a case-control design. Children aged 6 to 12 years with a clinical diagnosis of ADHD and TD controls were enrolled at Xinhua Hospital between January and May 2024. The primary objective was to conduct an initial evaluation of the diagnostic discrimination of BOKE STARS (BOKE Medical Technology Co Ltd) in distinguishing children with ADHD from controls. Secondary exploratory objectives were to examine differences across ADHD subtypes and to assess the relationships between BOKE STARS indices and parent-reported SNAP-IV symptom severity scores.

### Ethical Considerations

The study protocol was approved by the institutional review board of Shanghai Xinhua Hospital, Shanghai Jiao Tong University School of Medicine (XHEC-C-2024-012-2). Written informed consent was obtained from all legal guardians, and assent was obtained from participants aged ≥8 years after an age-appropriate explanation of the study procedures. Participant data were deidentified before analysis and stored on password-protected institutional systems accessible only to authorized study personnel. No financial compensation was provided for participation.

### Participants

#### Sample Size and Recruitment

Sample size was estimated using PASS software (version 15; NCSS LLC). On the basis of prior studies reporting an area under the receiver operating characteristic (ROC) curve (AUC) of ≥0.84 for similar attention-assessment devices, a target AUC of 0.95, α of .05 (2-sided), β of .20, and a 1:1 group ratio were assumed. After allowing for an anticipated dropout rate of 20%, the planned sample size was 100 participants (n=50 per group). A total of 100 participants were enrolled, including 50 (50%) children with ADHD and 50 (50%) TD controls. Children in the ADHD group were consecutively recruited from the Department of Developmental and Behavioral Pediatrics at Xinhua Hospital.

Control participants were recruited during the same study period through school-based recruitment from ordinary primary schools in Yangpu District, Shanghai, where Xinhua Hospital is located. The research team invited 14 primary schools in the district to participate, and 5 schools agreed to support recruitment. Study information was distributed to parents or legal guardians through the participating schools. After parental authorization was obtained, 50 children aged 6 to 12 years were randomly selected from 68 regular classes as candidate control participants and subsequently underwent eligibility screening before enrollment. Screening procedures included parent-reported medical and developmental history; parent-reported developmental, learning, or behavioral concerns; SNAP-IV symptom screening; and eligibility assessment by trained study personnel. TD controls were defined, based on parent-reported history and screening results, as children with no known previous or current diagnosis of ADHD or other neurodevelopmental or major psychiatric disorders, no clinically elevated ADHD-related symptoms identified through SNAP-IV screening, no parent-reported clinically significant developmental, learning, or behavioral concerns, and no neurologic disease, hearing impairment, or visual impairment.

#### Inclusion Criteria

General inclusion criteria were as follows: children aged 6 to 12 years, regardless of sex, who were able to complete study procedures and whose parents or legal guardians provided informed consent. Children aged ≥8 years also provided assent.

For the ADHD group, participants were required to have a clinical diagnosis of ADHD made by specialist clinicians in the Department of Developmental and Behavioral Pediatrics according to the *Diagnostic and Statistical Manual of Mental Disorders, fifth edition (DSM-5)*. Specialist clinical diagnosis according to DSM-5 criteria served as the diagnostic reference for ADHD group classification. All participants in the ADHD group were assessed before initiation of pharmacologic or nonpharmacologic treatment for ADHD.

#### Exclusion Criteria

Children were excluded if they had a history of neurological disease, severe head injury, intellectual disability, another major chronic illness, hearing or visual impairment that could interfere with audiovisual stimulus perception, long-term use of medications that could affect attention, arousal, or cognitive performance, or other major psychiatric disorders such as depression, anxiety, or psychosis. These exclusion criteria were intended to reduce major sources of neurocognitive confounding and to improve the interpretability of CPT performance.

Children in the ADHD group were additionally excluded if they had previously received or were currently receiving pharmacologic treatment for ADHD, including stimulant or nonstimulant ADHD medication, or structured nonpharmacologic interventions targeting ADHD symptoms, such as behavioral therapy, cognitive training, neurofeedback, or parent-management training, before the BOKE STARS assessment.

For the control group, children were additionally excluded if they had a known previous or current diagnosis of ADHD, clinically elevated ADHD-related symptoms suggested by SNAP-IV screening, autism spectrum disorder, specific learning disorder, tic disorder, parent-reported clinically significant developmental, learning, or behavioral concerns, or a first-degree relative diagnosed with ADHD.

### Measures

#### Swanson, Nolan, and Pelham Rating Scale–Fourth Version

The SNAP-IV was used to evaluate ADHD symptom severity based on parent report. The Chinese version of the SNAP-IV has shown satisfactory psychometric properties, including internal consistency and test-retest reliability, and has been used in Chinese clinical and research settings [15]. The scale contains 26 items across 3 subscales: inattention (items 1-9), hyperactivity or impulsivity (items 10-18), and oppositional defiant disorder (items 19-26). Each item is rated on a 4-point Likert scale from 0 (“not at all”) to 3 (“very much”), with higher scores indicating more severe symptoms.

Following the outpatient consultation, parents completed the SNAP-IV in a dedicated quiet evaluation room on a computer. Standardized instructions were provided by a trained evaluator, and parents completed the questionnaire independently. The evaluator was available to clarify procedural questions when needed. The main SNAP-IV outcomes recorded for analysis were the inattention score, hyperactivity or impulsivity score, oppositional defiant disorder score, 18-item total score, and 26-item total score. In this study, SNAP-IV was used as a caregiver-reported measure of symptom severity and was not used as the diagnostic reference standard.

#### BOKE STARS Assessment

BOKE STARS is a tablet-based bimodal CPT developed for Chinese children aged 6 to 12 years. The current version was developed by adapting the classic CPT framework to a pediatric audiovisual task format intended to better match children’s cognitive and behavioral characteristics in clinical assessment settings. During development, the BOKE STARS system underwent iterative technical testing and clinical usability refinement before the version used in this study was finalized. Internal testers and software developers evaluated system stability, stimulus presentation, response recording, and backend data output. Clinical feedback was obtained through pilot clinical use and consultation with pediatric clinicians, who provided recommendations on task parameters such as interstimulus and intertrial intervals, interface design, task instructions, and the practice procedure. Age-matched children also completed trial sessions to assess whether the task rules and response requirements were understandable and feasible. On the basis of this feedback, the task parameters, user interface, standardized instructions, practice phase, and data-output procedures were adjusted to improve usability and feasibility in pediatric assessment settings. Additional details on consultation participants, user testing, feedback categories, iteration rounds, and modifications between iterations are provided in Multimedia Appendix 1.

**Figure 1.**
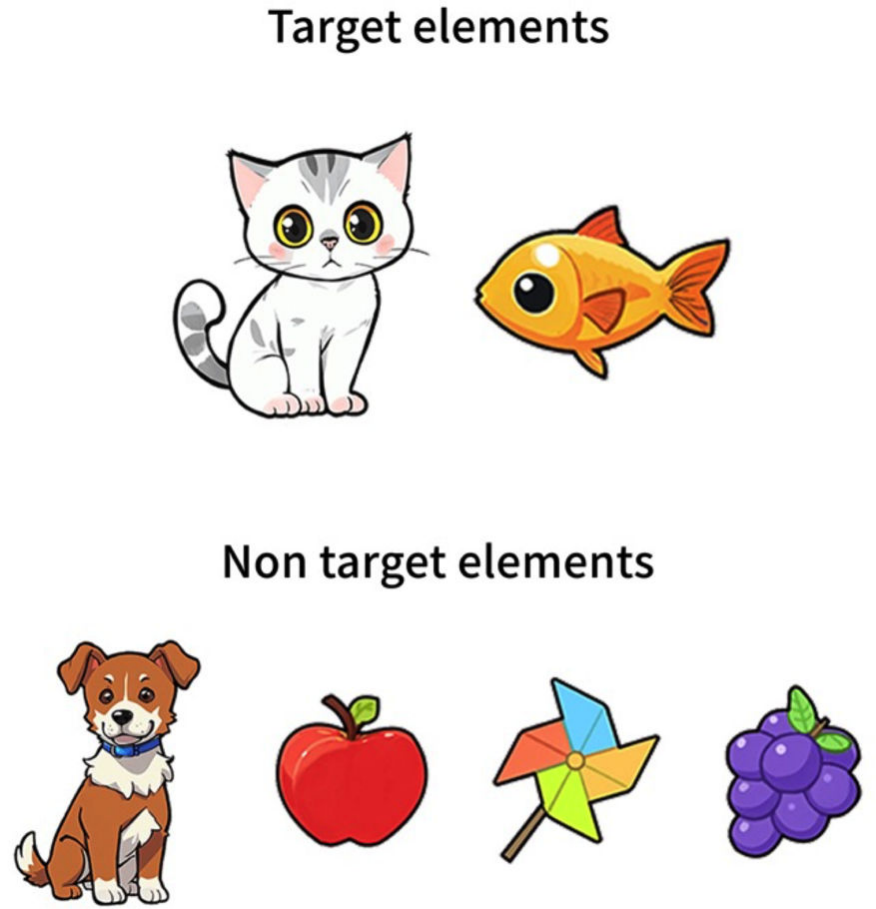
Representative interface and stimulus of BOKE STARS. The tablet-based task presents visual, auditory, and audiovisual stimuli. Participants respond to target stimuli and withhold responses to nontarget stimuli. Target trials include a cat image paired with a fish image or a cat meow paired with a fish image; nontarget trials include other auditory or visual stimuli. The task generates standardized indices, including omission errors, commission errors, reaction time, reaction time variability, discrimination prime, and total score.

In this study, BOKE STARS was administered on a tablet device in a quiet room, with assistance from a trained evaluator throughout the assessment. The task duration was approximately 12.5 minutes. Participants were instructed to press a response button as quickly as possible when a target stimulus appeared and to withhold responses to nontarget stimuli. The target condition consisted of the simultaneous presentation of a cat image and a fish image, or a cat meow paired with a fish image (Figure 1). Nontarget stimuli included other auditory or visual stimuli, such as a dog bark, bones, grapes, or windmills (Figure 1).

The test comprised 8 blocks, each containing 36 stimuli. Stimulus presentation varied by target-to-nontarget ratio and by stimulus modality (visual, auditory, or audiovisual). Stimuli were presented randomly in the center of the screen for 250 ms at interstimulus intervals of 1.5 s or 3 s. Before formal testing, all participants completed a practice phase and mock trials to confirm task understanding.

After task completion, the system generated standardized scores for the key indices used in this study: omission errors, commission errors, reaction time, RTV, discrimination prime, and total score. In this scoring framework, lower standardized scores indicated poorer performance.

### Administration Procedure

Both SNAP-IV and BOKE STARS were completed during the same study visit under standardized conditions. Following routine clinical consultation, parents completed the SNAP-IV in a dedicated quiet evaluation room with standardized instructions provided by trained study personnel. Children then underwent the BOKE STARS assessment individually in a quiet room under evaluator supervision. Parental or guardian presence was not required during formal BOKE STARS administration. After informed consent had been obtained from parents or legal guardians, each child completed the task with guidance and monitoring from a trained evaluator, but without assistance from accompanying family members during task performance.

Children in the ADHD group were diagnosed by specialist clinicians in the Department of Developmental and Behavioral Pediatrics at enrollment or during the recent pre-enrollment clinical evaluation and had not yet received pharmacologic or nonpharmacologic treatment for ADHD at the time of study assessment. This design was intended to reduce the potential confounding effects of prior intervention on performance-based outcomes.

### Outcomes

The primary outcome of the study was the diagnostic discrimination of key BOKE STARS indices, assessed primarily through ROC analysis. Group comparisons of BOKE STARS indices between the ADHD and control groups were treated as supportive analyses. ADHD subtype comparisons and correlations between BOKE STARS indices and SNAP-IV scores were predefined as secondary exploratory analyses.

After each BOKE STARS assessment, the evaluator uploaded the test data, and the system backend generated a CSV summary file. The indices analyzed in this study were omission errors, commission errors, reaction time, RTV, discrimination prime, and total score.

### Statistical Analysis

Statistical analyses were performed using SPSS (version 26.0; IBM Corp). Descriptive statistics were used to summarize demographic and clinical characteristics. Data normality was evaluated using the Shapiro-Wilk test in combination with histogram inspection. Continuous variables with normal distributions are reported as mean (SD), whereas nonnormally distributed variables are reported as median (IQR). Categorical variables are reported as n (%).

Group differences between the ADHD and control groups were analyzed using independent-samples *t* tests (2-tailed) or Mann-Whitney *U* tests for continuous variables and chi-square tests for categorical variables. ADHD subtype comparisons were performed using 1-way ANOVA or Kruskal-Wallis tests, as appropriate. Spearman rank correlation analysis was used to assess associations between SNAP-IV scores and BOKE STARS indices.

Diagnostic discrimination was evaluated using ROC curve analysis in the full case-control sample. AUCs and 95% CIs were calculated for each BOKE STARS index. Sensitivity and specificity were reported descriptively across ROC-derived cutoffs to illustrate the tradeoff between sensitivity and specificity across the full ROC curve. All tests were 2-sided, and *P*<.05 was considered statistically significant.

## Results

### Participant Characteristics

**Figure 2.**
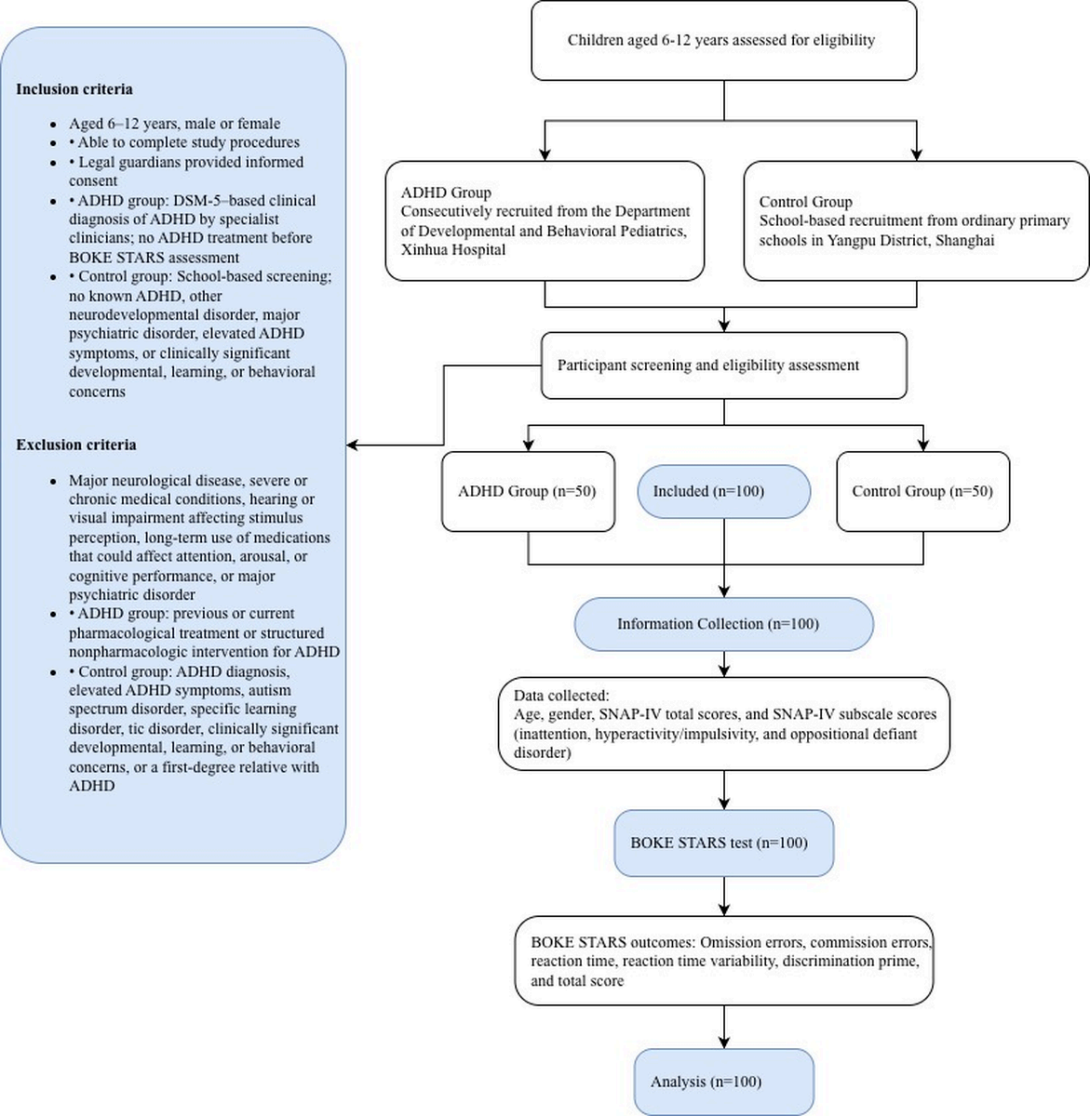
Study flow and assessment procedures in a prospective single-center case-control diagnostic accuracy study of BOKE STARS for attention-deficit/hyperactivity disorder (ADHD) assessment in Chinese children aged 6 to 12 years at Xinhua Hospital, Shanghai, China. SNAP-IV: Swanson, Nolan, and Pelham Rating Scale–fourth version.

The study design is summarized in Figure 2.

A total of 100 children were included in the analysis: 50 children with ADHD and 50 TD controls. The median age was 8.0 (IQR 7.00-9.00) years in both groups. The ADHD group included a higher proportion of boys than the control group (n=82, 82% vs n=40, 40%*; χ*^2_1_^=18.5; *P*<.001), which is broadly consistent with prior epidemiological findings [14,15]. As expected, the ADHD group had significantly higher SNAP-IV inattention, hyperactivity, or impulsivity, 18-item total, and 26-item total scores than the control group (Table 1).

Within the ADHD group, subtype distribution was as follows: inattentive type, 42% (n=42); hyperactive or impulsive type, 18% (n=18); combined type, 34% (n=34); and not otherwise specified type, 6% (n=6).

Compared with controls, children with ADHD showed significantly poorer performance across all major BOKE STARS indices, including omission errors, commission errors, reaction time, RTV, discrimination prime, and total score (all *P*≤.01; Table 2).

**Table 1.** Baseline demographic and clinical characteristics of the attention-deficit/hyperactivity disorder (ADHD) and control groups.

Variables	ADHD group (n=50)	Control group (n=50)	Test statistic^a^	*P* value
Age (years), median (IQR)	8.00 (7.00-9.00)	8.00 (7.00-10.00)	−0.598	.55
Age (months), median (IQR)	98.50 (89.50-115.25)	105.00 (87.75-122.50)	−0.528	.60
Sex (male), n (%)	41 (82)	20 (40)	18.537 (1)	<.001
SNAP-IV^b^ inattention score, median (IQR)	8.00 (6.00-13.00)	6.00 (3.25-7.00)	−4.895	<.001
SNAP-IV hyperactivity or impulsivity score, median (IQR)	6.00 (3.75-10.00)	3.00 (1.00-5.25)	−4.629	<.001
SNAP-IV oppositional defiant disorder, median (IQR)	4.00 (2.00-8.00)	4.00 (2.00-6.00)	−0.869	.38
SNAP-IV 18-item total score, median (IQR)	13.00 (11.00-26.25)	9.00 (5.50-12.25)	−5.042	<.001
SNAP-IV 26-item total score, median (IQR)	17.50 (13.00-31.50)	14.00 (7.00-18.25)	−3.999	<.001

a For categorical variables reported as n (%), the test statistic represents the *χ*2 (*df*). For continuous variables reported as median (IQR), the test statistic represents the Mann-Whitney *U* test Z value.

b SNAP-IV: Swanson, Nolan, and Pelham Rating Scale–fourth version.

**Table 2.** Comparison of BOKE STARS indices between the attention-deficit/hyperactivity disorder (ADHD) and control groups.

Variables	ADHD group (n=50)	Control group (n=50)	Test statistic^a^	*P* value
Omission errors, median (IQR)	83.00 (74.00-92.00)	102.00 (99.25-110.00)	−6.682	<.001
Commission errors, median (IQR)	97.00 (81.75-103.00)	103.50 (96.75-116.00)	−3.259	.001
Reaction time, mean (SD)	92.38 (18.01)	100.72 (13.40)	2.627 (49)	.01
Reaction time variability, mean (SD)	80.08 (13.45)	103.32 (11.50)	9.287 (49)	<.001
Discrimination prime, median (IQR)	89.50 (78.00-98.00)	104.00 (98.00-114.00)	−5.495	<.001
Total score, median (IQR)	353.00 (332.25-377.25)	415.00 (393.75-427.25)	−7.960	<.001

a For continuous variables reported as median (IQR), the test statistic represents the Mann-Whitney *U* test *Z* value. For continuous variables reported as mean (SD), the test statistic represents the independent-samples *t* test (*df*) value.

### Primary Diagnostic Accuracy Analysis

**Figure 3.**
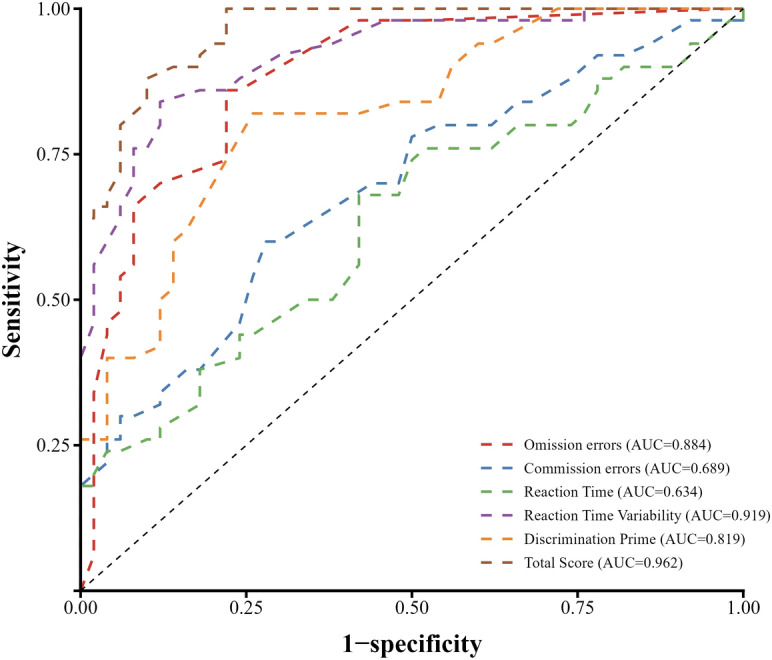
Receiver operating characteristic curves for BOKE STARS indices in the full case-control sample. Curves are shown for omission errors, commission errors, reaction time, reaction time variability, discrimination prime, and total score in distinguishing children with attention-deficit/hyperactivity disorder (ADHD) from typically developing controls. Total score and reaction time variability showed the strongest discriminatory performance.

ROC analyses were performed to assess the diagnostic discrimination of the major BOKE STARS indices in the full case-control sample (Figure 3). The total score showed the highest diagnostic discrimination, with an AUC of 0.962 (95% CI 0.931-0.992), followed by RTV (AUC 0.919, 95% CI 0.867-0.971) and omission errors (AUC 0.884, 95% CI 0.818-0.950). Discrimination prime showed moderate discrimination (AUC 0.819, 95% CI 0.737-0.900), whereas commission errors (AUC 0.689, 95% CI 0.585-0.792) and reaction time (AUC 0.634, 95% CI 0.524-0.743) showed comparatively weaker discrimination.

Sensitivity and specificity across ROC-derived cutoffs are shown in Figure 3. For the total score, descriptive cutoffs illustrated different sensitivity-specificity tradeoffs; for example, a cutoff of ≤394.5 yielded a sensitivity of 1.00 and specificity of 0.72, whereas a cutoff of ≤385.5 yielded a sensitivity of 0.90 and specificity of 0.86. For RTV, sensitivity and specificity similarly varied across cutoffs; for example, a cutoff of ≤96.5 yielded a sensitivity of 0.88 and specificity of 0.76, whereas a cutoff of ≤91.5 yielded a sensitivity of 0.80 and specificity of 0.88. These values are presented to describe ROC performance in this sample rather than to establish clinically validated diagnostic thresholds.

Overall, the full-sample ROC results suggested that the total score and RTV were the strongest discriminatory indices among the BOKE STARS measures. However, because this study used a case-control design with an artificially fixed ADHD-to-control ratio, the AUC, sensitivity, specificity, and threshold estimates should be interpreted as preliminary indicators of diagnostic discrimination and may not reflect real-world clinical diagnostic performance.

BOKE STARS showed promising preliminary diagnostic discrimination for identifying Chinese children with ADHD in this case-control sample, with the total score and RTV showing the strongest discriminatory performance. However, because the case-control design artificially fixed the ratio of ADHD cases to controls, diagnostic performance estimates and exploratory cutoffs should be interpreted cautiously and should not be considered representative of real-world clinical diagnostic performance. BOKE STARS may serve as an adjunctive assessment tool to complement clinical interviews and caregiver-reported rating scales, but further external validation in larger and clinically heterogeneous populations is required before broader clinical implementation.

### Exploratory ADHD Subtype Analysis

Exploratory comparisons across ADHD subtypes did not reveal statistically significant differences for any BOKE STARS index (all *P*>.05; Table 3). These analyses should be interpreted cautiously because subgroup sizes were small and uneven, especially for the not otherwise specified subtype.

**Table 3.** Exploratory comparison of BOKE STARS indices across attention-deficit/hyperactivity disorder (ADHD) subtypes.

Variables	Inattentive (n=21)	Hyperactive or impulsive (n=9)	Combined (n=17)	Not otherwise specified (n=3)	*P* value
Omission errors, median (IQR)	81.00 (74.00-87.50)	80.00 (74.50-100.00)	87.00 (80.00-91.00)	53.00 (53.00-76.50)	.49
Commission errors, median (IQR)	96.00 (76.50-102.00)	97.00 (91.50-100.00)	98.00 (82.00-111.50)	103.00 (101.50-103.00)	.51
Reaction time, mean (SD)	97.67 (17.78)	86.11 (12.86)	92.65 (17.47)	72.67 (25.15)	.08
RTV, mean (SD)	80.14 (14.50)	77.56 (12.64)	83.53 (11.88)	67.67 (14.05)	.27
Discrimination prime, median (IQR)	89.00 (76.50-94.50)	97.00 (82.50-98.00)	96.00 (82.00-102.00)	81.00 (77.50-92.00)	.54
Total score, median (IQR)	350.00 (325.00-377.00)	352.00 (318.50-378.00)	367.00 (337.00-378.00)	289.00 (274.00-337.00)	.68

### Exploratory Correlation Analysis

Several BOKE STARS indices were modestly negatively correlated with SNAP-IV inattention and hyperactivity or impulsivity scores (Table 4). Omission errors were negatively correlated with inattention (ρ=−0.327; *P*=.001), hyperactivity or impulsivity (ρ=−0.230; *P*=.02), and the 18-item total score (ρ=−0.296; *P*=.003). RTV was negatively correlated with inattention (ρ=−0.390; *P*<.001), hyperactivity or impulsivity (ρ=−0.354; *P*<.001), the 18-item total score (ρ=−0.386; *P*<.001), and the 26-item total score (ρ=−0.336; *P*<.001).

**Table 4.** Exploratory correlations between the Swanson, Nolan, and Pelham Rating Scale–fourth version (SNAP-IV) scores and BOKE STARS indices.

SNAP-IV domain	Omission errors	Commission errors	Reaction time	Reaction time variability	Discrimination prime	Total score
Inattention	−0.327^a^	−0.202^b^	−0.147	−0.390^a^	−0.300^a^	−0.387^a^
Hyperactivity or impulsivity	−0.230^b^	−0.175	−0.101	−0.354^a^	−0.226^b^	−0.339^a^
Oppositional defiant disorder	0.138	−0.003	0.081	−0.136	0.099	0.055
18-item total	−0.296^a^	−0.191	−0.140	−0.386^a^	−0.271^a^	−0.379^a^
26-item total	−0.165	−0.124	−0.088	−0.336^a^	−0.152	−0.257^a^

a *P*<.01.

b *P*<.05.

The total score was negatively correlated with inattention (ρ=−0.39; *P*<.001), hyperactivity or impulsivity (ρ=−0.34; *P*=.001), the 18-item total score (ρ=−0.379; *P*<.001), and the 26-item total score (ρ=−0.257; *P*<.001). No significant correlations were found between oppositional defiant disorder scores and BOKE STARS indices.

## Discussion

### Principal Findings

In alignment with our primary objective to evaluate the diagnostic discrimination of the BOKE STARS bimodal CPT in a Chinese clinical setting, our results demonstrate that children with ADHD performed significantly worse than TD controls across all major performance indices. In the full case-control sample, the total score showed the strongest overall diagnostic discrimination, followed by RTV and omission errors. Regarding our secondary exploratory aims, we found no significant differences in BOKE STARS indices across ADHD subtypes and observed only modest correlations between objective performance indices and subjective SNAP-IV symptom ratings.

These findings suggest that BOKE STARS may capture objective differences in sustained attention, response control, and reaction time stability between children with ADHD and TD controls. The relatively strong discriminatory performance of RTV is broadly consistent with prior literature suggesting that RTV is an important feature of ADHD-related attentional instability [16,17].

The modest correlation between BOKE STARS indices and SNAP-IV scores is consistent with previous research indicating that objective CPT performance and caregiver-reported behavioral ratings capture overlapping but distinct constructs [7,18]. Rating scales summarize caregiver observations across everyday contexts, whereas CPTs capture task-based performance under standardized testing conditions. In addition, the findings suggest that a bimodal audiovisual CPT paradigm may be feasible and informative in this clinical context. However, determining whether such a format offers advantages over more traditional unimodal paradigms will require direct comparative evaluation in future studies.

Several limitations must be considered when interpreting these results, particularly regarding potential biases in diagnostic accuracy estimates. First, the case-control design may have led to overestimation of diagnostic accuracy. By excluding more diagnostically ambiguous or clinically complex children, such as those with subthreshold symptoms or comorbid conditions that may mimic ADHD, and instead comparing clinically identified ADHD cases with TD controls, this study may have inflated estimates of AUC, sensitivity, and specificity. In addition, the fixed 1:1 ratio of ADHD cases to controls does not reflect the true prevalence of ADHD in the target clinical population. Therefore, the ROC-derived cutoff values reported in this study should be interpreted only as exploratory descriptions of sensitivity-specificity tradeoffs within this sample and should not be considered validated clinical diagnostic thresholds. Similar design-related inflation in diagnostic test performance has been described in the methodological literature, particularly in studies that include clearly defined cases and healthy controls [19,20]. Second, knowledge of diagnosis and temporal factors may have introduced bias. The duration of the ADHD diagnosis and parents’ and children’s awareness of the condition may have influenced caregiver-reported SNAP-IV ratings or children’s motivation and test anxiety during the BOKE STARS assessment.

Third, the role of SNAP-IV in this study should be interpreted cautiously. Although the Chinese parent version of the SNAP-IV has demonstrated acceptable psychometric validity and has been used in Chinese clinical and research settings [15], SNAP-IV remains a caregiver-reported symptom rating scale rather than a diagnostic reference standard. Therefore, correlations between SNAP-IV scores and BOKE STARS indices should be interpreted as associations between parent-reported symptoms and task-based performance, rather than as evidence of diagnostic equivalence. Finally, sample characteristics and sample stability also limit interpretation. The study’s single-center nature, small sample size, and significant sex imbalance in the ADHD group limit the generalizability of the findings. Additionally, the lack of test-retest reliability data means we cannot yet confirm the temporal stability of the BOKE STARS indices in this population.

Despite these limitations, this study provides preliminary evidence that BOKE STARS may serve as a useful adjunctive tool in the assessment of Chinese children undergoing ADHD evaluation. Future work should prioritize external validation in larger and more clinically heterogeneous samples, direct comparisons with established CPT systems, evaluation of test-retest reliability, and a more rigorous examination of the incremental clinical value provided by BOKE STARS when it is added to standard diagnostic workflows.

### Conclusions

The results of this study suggest that BOKE STARS may serve as a promising adjunctive tool that provides an objective layer of data within the traditional ADHD diagnostic workflow in China. However, its role should be viewed as complementary to, rather than a replacement for, clinical interviews, *DSM-5*–based clinical diagnosis, caregiver-reported rating scales, and longitudinal behavioral observations. The ROC-derived results and cutoff values reported here should be interpreted as preliminary case-control findings rather than validated indicators of real-world diagnostic performance.

More broadly, these findings support continued investigation of performance-based digital assessment tools as part of multimodal ADHD evaluation. External validation in independent, larger, and clinically heterogeneous populations of children undergoing real-world ADHD assessment is needed before the findings can be generalized to routine clinical diagnostic settings. Further work should determine the generalizability, temporal stability, and incremental clinical value of bimodal CPTs such as BOKE STARS within standard diagnostic workflows.

## Supplementary material

10.2196/82164 Multimedia Appendix 1 Development and usability refinement process of BOKE STARS (Sustained Task and Attention Response Screening).

## Abbreviations

ADHD: attention-deficit/hyperactivity disorder
AUC: area under the receiver operating characteristic curve
CPT: continuous performance test
*DSM-5*: *Diagnostic and Statistical Manual of Mental Disorders, fifth edition*
ROC: receiver operating characteristic
RTV: reaction time variability
SNAP-IV: Swanson, Nolan, and Pelham Rating Scale–fourth version
STARS: Sustained Task and Attention Response Screening
TD: typically developing
