# Supplementary material for: Diagnostic Discrimination of BOKE STARS, a Bimodal Continuous Performance Test, for Attention-Deficit/Hyperactivity Disorder Assessment in Chinese Children: Single-Center Case-Control Study
Source: JMIR Form Res. 2026 Jul 21;10:e82164. doi: 10.2196/82164 (PMC13387741; doi:10.2196/82164)
Supplement: Multimedia Appendix 1 [file formative-v10-e82164-s001.docx]

**Multimedia Appendix X. Development and usability refinement process of BOKE STARS**

BOKE STARS was developed through a structured, iterative process to adapt a classic continuous performance test framework to a pediatric bimodal audiovisual format suitable for Chinese children aged 6 to 12 years. The audiovisual format was selected because traditional CPTs commonly rely on unimodal visual or auditory stimuli, whereas children with ADHD may show difficulties in sustained attention, response inhibition, and reaction time stability. The bimodal audiovisual format was intended to improve child engagement and task ecological relevance while preserving the core CPT requirements of sustained attention and response inhibition.

The development and usability refinement process included 4 iterative rounds: internal technical testing, multidisciplinary clinical consultation, child user testing, and finalization of the study version. Internal testing involved 2 product team members, 7 medical researchers, 9 research and development personnel, and 2 testers. Clinical consultation involved 19 experts and clinicians from developmental-behavioral pediatrics, psychiatry and psychology, and general pediatrics. User testing was completed by 33 children aged 6 to 12 years who were invited through clinical recommendation with parental consent or through internal employee families. These children were not included in the formal study sample. User testing was conducted to evaluate whether children could understand the task rules through automated instructions, complete the practice phase, operate the response procedure smoothly, distinguish the interface and stimulus materials, and tolerate the task procedure.

The version used in the formal study was finalized before participant enrollment. During the study period, all participants completed the same version of BOKE STARS, and no changes were made to task parameters, stimulus presentation, scoring rules, assessment procedures, or data-output procedures. Experts involved in usability refinement did not participate in the subsequent clinical study.

| Iteration Round | Participants involved | Purpose | Feedback received | Modification made |
| --- | --- | --- | --- | --- |
| Round 1: Testing technical testing | 2 product team members, 7 medical researchers, 9 research and development personnel, and 2 testers | To evaluate system stability, task logic, stimulus presentation, response recording, and backend data output | Feedback focused on system stability, timing accuracy, response recording, backend CSV output, and operational workflow | Optimized stimulus presentation, response recording, backend data export, task-flow logic, and system stability |
| Round 2: Multidisciplinary clinical consultation | 19 experts and clinicians from developmental-behavioral pediatrics, psychiatry and psychology, and general pediatrics | To evaluate clinical feasibility and suitability for children aged 6 to 12 years | Feedback focused on task comprehensibility, task duration, interstimulus and intertrial intervals, interface design, instructions, and practice procedures | Refined task parameters, adjusted interface layout, standardized task instructions, and optimized the practice phase |
| Round 3: Child user testing | 33 children aged 6 to 12 years; all were outside the formal study sample | To evaluate whether children could understand the rules, complete practice trials, operate the task smoothly, and distinguish stimulus materials | Feedback focused on rule comprehension, operational feasibility, practice-phase adequacy, stimulus distinguishability, and tolerance of the task procedure | Improved automated instructions, clarified practice procedures, refined visual and auditory prompts, and adjusted operational guidance |
| Round 4: Finalization of study version | Research, medical, product, and development teams | To integrate feedback and finalize the version used in the formal study | Final review focused on consistency of task parameters, scoring rules, assessment procedures, and data-output procedures | Fixed the formal study version before participant enrollment; no changes were made during the study period |
